# Quantitative NMR Interpretation without Reference

**DOI:** 10.1155/2022/7490691

**Published:** 2022-11-10

**Authors:** Priscila Ivo Rubim de Santana, Joyce Sobreiro Francisco Diz de Almeida, Tanos Celmar Costa França, Jochen Junker

**Affiliations:** ^1^Laboratory of Molecular Modeling Applied to Chemical em Biological Defense (LMCBD), Military Institute of Engineering, Rio de Janeiro 22290-270, Brazil; ^2^Oswaldo Cruz Foundation, CDTS, Av. Brasil 4365, Rio de Janeiro 21040-900, Brazil; ^3^Department of Chemistry, Faculty of Science, University of Hradec Kralove, Rokitanskeho 62, 500 03 Hradec Kralove, Czech Republic

## Abstract

As has been documented numerous times over the years, nuclear magnetic resonance (NMR) experiments are intrinsically quantitative. Still, quantitative NMR methods have not been widely adopted or largely introduced into pharmacopoeias. Here, we describe the quantitative interpretation of the 1D proton NMR experiment using only absolute signal intensities with the variation of common experimental parameters and their application.

## 1. Introduction

Since its inception, NMR has always been considered inherently quantitative [1–6] and it has been used in teaching [7]. As opposed to all other spectroscopic methods, the intensity of an NMR signal is directly proportional to the abundance of the nuclei causing it [6–8], which could even be in multiple molecules [9, 10]. In the case of simple mixtures, NMR allows for simultaneous quantification of the constituents based on one sole reference standard. The standard does not have to share its identity with any of the analytes of interest. This key feature makes quantitative NMR an extremely versatile technique, and numerous applications for the quantitative analysis of pharmaceutical compounds have been proposed over the decades [6, 11–23]. The majority of the described experiments are 1D liquid state, but 2D and CPMAS experiments have also been proposed. Also, most of the proposed quantitative methods are based on proton NMR experiments, but other nuclei have been used since the beginning: ^31^P [24, 25], ^2^H [26], and ^13^C [19, 27, 28]. In recent years, ^23^Na [29], ^19^F [30, 31], ^35^Cl [32, 33], ^11^B [34], ^7^Li [35], and quadrupolar nuclei like ^27^Al [36, 37] and ^14^N [38, 39] were added to the list. While some of the proposed methods are 2D experiments or CPMAS, mostly 1D liquid state experiments have been described.

Whichever method is chosen, the quantification by NMR is always based on the comparison of the signal intensity of reference material with the signal intensity of the analyte(s), as the intensities are proportional to the molar concentrations and the number of protons contributing to the signal. The reference signal can be provided by a reference material mixed with the analyte in one solution, internal referencing (IR) [18, 25, 29, 30, 32, 40–44], or by a separate solution, external reference (ER). Two methods for ER have been described; most commonly, two identical experiments are carried out, one time with the analyte and the other time with the reference material [6, 45, 46]. Alternatively, a solution with the reference is sealed into a capillary that is then added to the solution of the analyte [47]. Hybrid methods like ERETIC [48–50] and PULCON [46, 51–56] have also been implemented, which combine ER and IR by an intermediate step. All these methods work with the best reliability when the reference used has a molar concentration that is close to the analyte's concentration, thus requiring some previous knowledge about the analyte. The analysis of mixtures can also be restricted, as the quantification reliability might vary with the concentrations involved.

Several experimental parameters shown in Table 1 have influence on the NMR spectrum, and some of them are flexible depending on the chosen method. Here, we demonstrate a new hybrid method, flexible absolute intensity-based quantification by NMR (FAINT-NMR), which can be applied to the quantification of compounds, even with largely varying concentrations, without previous knowledge. The work presented demonstrates that the restrictions described for external referencing methods [46] are not necessary. The normalization of the absolute signal intensity by a receiver gain and the number of scans results in an Intensity Gain (IG) factor, based on which the quantification of every sample becomes possible, independent of the experimental parameters. As amplifiers are notoriously nonlinear, a manual linearization of the receiver gain values was performed, in order to verify if this would improve the quantification quality further.

## 2. Experimental

The usability of FAINT-NMR was verified on a Bruker equipment AVANCE III 400 MHz equipped with a 5 mm BBO Prodigy probe and a sample changer, which was used with as much automation as possible for experiment acquisition, followed by partially automated interpretation. As the methods target small molecules, protons were chosen as the observed nucleus due to higher sensitivity and sufficient signal separation.

Samples were weighted on a calibrated Mettler Toledo AG245 balance and diluted with 0.6 ml of DMSO-d_6_ into 5 mm NMR tubes. After determining values for the fixed parameter (D1), the influence of the flexible parameters (NS, RG) was determined. The longest *T*_1_ of the reference material was determined as 2.06 s by our own measurements in DMSO-d_6_, as 1.86 s in CDCl_3_ [57] and 2.7 s in D_2_O [58]; thus, the inter-scan delay D1 was fixed as 16 s for all experiments.

Simple proton experiments with a 90° pulse and 16 k observe points were obtained at 25°C, varying the number of scans and receiver gain. Experiments with 2 to 64 scans (NS) and receiver gain (RG) from 25.4 to the highest RG value determined by the function automatic receiver gain (RGA) for the sample were carried out in duplicates. The RG values available on the equipment usually reach values above 4 K, which we could not observe for our samples for proton experiments. For proton experiments, we observe that the maximum RG value for low analyte concentrations is actually defined by the solvent “concentration” in the sample. When the analyte is in high concentration, it can decrease the RG value, as seen. Thus, the experiments use only a small slice of the possible RG values. All experiments were processed automatically (Fourier transformation and phase correction), followed by integration using intervals defined on one reference experiment. With this data set a constant IG (Intensity Gain, *I∗NS*^−1^*∗RG*^−1^*∗*[mMol]^−1^) factor was determined, that allows the calculation of the concentration directly from the absolute intensity of a signal. Finally, a linearization of the RG values was carried out, and the improvement of the back-calculated values was verified.

## 3. Results

**Figure 1:**
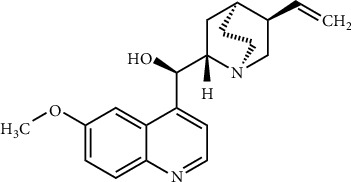
Quinine (**1**, C_20_ H_24_ N_2_ O_2_, MW 324.42 g/mol), used for the qNMR application.

**Figure 2:**
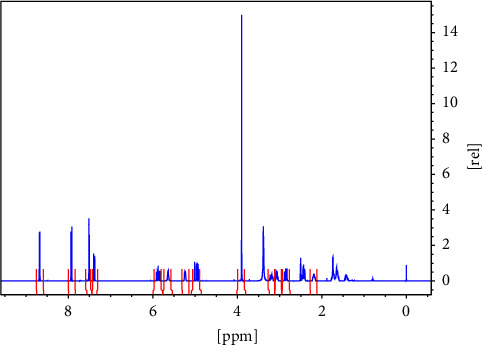
^1^H NMR spectrum of Quinine (**1**), the used integration limits are delimited. Further signals were not used due to overlap and complex coupling patterns.

FAINT-NMR was applied to a series of quinine samples, Figure 1, which was chosen due to its high molecular weight.  Figure 2 shows the proton NMR spectrum of quinine in DMSO-d_6_ and the signals that were used for the quantification. Further signals were not used because they overlap or have complex multiplet patterns. In total, five samples diluted in 0.6 ml of DMSO-d_6_ were used, as shown in Table 2.

**Figure 3:**
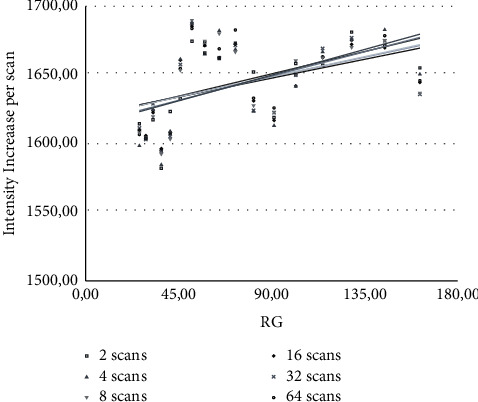
The average signal intensity increment was observed in dependence on sample concentration, the number of protons, and scans, separated for different RG values.

In Figure 3, the absolute signal intensities of 13 signals of quinine were averaged, normalized against their concentration, number of protons and scans, and scatter-plotted according to their respective RG. These signals were chosen because of their lack of overlap and the small number of observed couplings. The signal-to-noise ratio of all signals was always above 200 : 1.

The results from Figure 3 show that the per-scan signal intensity increment scatters around an average of 1650. Based on this, an IG factor of 1650 was defined for all intensity-based quantifications shown here. Furthermore, these results were used to carry out a manual linearization of the RG values, to further improve the results. The original and linearized RG values are shown in Table 3.

**Figure 4:**
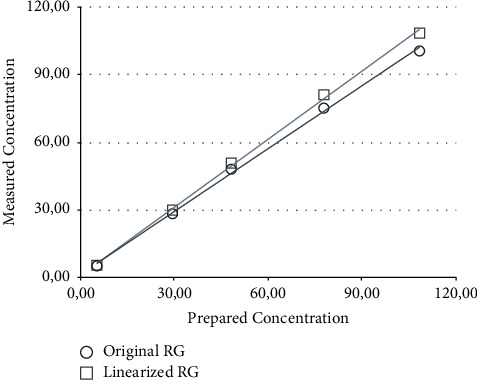
Back-calculated sample concentrations using not linearized RG values (original RG) and manually linearized RG values (linearized RG) plotted against the prepared concentrations.

In Table 4, the 5 actual sample concentrations are compared to the back-calculated values (BC) and values back-calculated using a linearized RG (BC-l).  Figure 4 shows the linear regression graph of the values in Table 4. The linear regression equations in Table 5 clearly show that the linearization of the RG improves the results, as the slope for the equation is very close to the optimum value of 1.0.

## 4. Conclusions

So far, a large-scale application of qNMR has been restricted by experimental conditions. In the case of internal reference methods, difficulties might arise because of signal overlap or interaction of the reference with the sample. In the case of external referencing, the fixed experimental conditions usually restrict the working range of the method. The results presented here show that some experimental parameters, like RG and NS, can be varied largely without affecting the quality of the quantification result. The linearization of the RG values further improves the accuracy of the method. By lifting these restrictions, FAINT-NMR can facilitate the quantification by NMR in general, including trace amounts in samples, as long as well-isolated signals are observed. One possibility to achieve theses isolated signals would be to combine Bayesian data analysis with FAINT-NMR, which would provide isolated signals and turn integration limits unneccessary.

## Figures and Tables

**Table 1:** Flexibility of the parameters that influence an NMR spectrum for quantitative applications.

Parameter	Influence on spectrum	IR	ER	FAINT-NMR
Temperature (T)	Signal position and intensity	Not recommended	No	Not recommended
Delay between scans (D1)	Signal intensity	No	No	No
Number of scans (NS)	Signal intensity and SN	Yes	No	Yes
Receiver gain (RG)	Signal intensity	Yes	No	Yes

IR, internal referencing; ER, external referencing; SN, signal-to-noise relation.

**Table 2:** Samples of prepared quinine and the count of possible RG values and the highest RG value for each sample.

Sample	Tc (mMol)	*W* (mg)	RC (mMol)	possible RG values	highest RG value
1	5	1.03	5.29	17	161
2	30	5.73	29.44	12	90.5
3	50	9.38	48.19	12	90.5
4	80	15.14	77.78	5	40.3
5	110	21.09	108.35	4	36

Tc, target concentration; *W*, real weight used; RC, real concentration; RG, receiver gain.

**Table 3:** Original RG values and linearized RG values.

Original	Linearized
25.4	23.58
28.5	26.35
32	29.99
36	33.07
40.3	37.35
45.2	43.15
50.8	49.45
57	55.05
64	62.05
71.8	69.60
80.6	76.10
90.5	85.10
101	96.80
114	109.80
128	124.00
144	140.00
161	153.00

**Table 4:** Back-calculated sample concentrations using not linearized RG values (BC) and manually linearized RG values (BC-l).

Sample	RC (mMol)	BC (mMol)	*σ*	BC-l (mMol)	*σ*
1	5.29	5.35	0.10	5.63	0.02
2	29.44	28.43	0.58	30.05	0.05
3	48.19	48.10	0.98	50.84	0.06
4	77.78	75.14	0.53	81.05	0.13
5	108.35	100.42	0.75	108.30	0.15

RC, real concentration; BC, back-calculated concentration; BC-l, back-calculated concentration with linearized RG; *σ*, Standard deviation.

**Table 5:** Linear regression equations for Table 4 and correlation factors.

RG	Linear Regression	*R* ^2^
Native	*y*=0.9275*x*+1.5808	0.9983
Linearized	*y*=1.0046*x*+1.1181	0.9987

## Data Availability

All data (NMR data as raw, processed, and integrated; Spreadsheet with data interpretation) are available at https://doi.org/10.5281/zenodo.7221753.
